# Transcriptional Regulation of Human Dual Specificity Protein Phosphatase 1 (DUSP1) Gene by Glucocorticoids

**DOI:** 10.1371/journal.pone.0013754

**Published:** 2010-10-29

**Authors:** Lauren E. Shipp, Joyce V. Lee, Chi-Yi Yu, Miles Pufall, Pili Zhang, Donald K. Scott, Jen-Chywan Wang

**Affiliations:** 1 Department of Nutritional Science and Toxicology, University of California, Berkeley, California, United States of America; 2 Department of Cellular and Molecular Pharmacology, University of California San Francisco, San Francisco, California, United States of America; 3 Division of Endocrinology, Department of Medicine, University of Pittsburgh, Pittsburgh, Pennsylvania, United States of America; Comprehensive Pneumology Center, Germany

## Abstract

**Background:**

Glucocorticoids are potent anti-inflammatory agents commonly used to treat inflammatory diseases. They convey signals through the intracellular glucocorticoid receptor (GR), which upon binding to ligands, associates with genomic glucocorticoid response elements (GREs) to regulate transcription of associated genes. One mechanism by which glucocorticoids inhibit inflammation is through induction of the dual specificity phosphatase-1 (DUSP1, a.k.a. mitogen-activated protein kinase phosphatase-1, MKP-1) gene.

**Methodology/Principal Findings:**

We found that glucocorticoids rapidly increased transcription of DUSP1 within 10 minutes in A549 human lung adenocarcinoma cells. Using chromatin immunoprecipitation (ChIP) scanning, we located a GR binding region between −1421 and −1118 upstream of the DUSP1 transcription start site. This region is active in a reporter system, and mutagenesis analyses identified a functional GRE located between −1337 and −1323. We found that glucocorticoids increased DNase I hypersensitivity, reduced nucleosome density, and increased histone H3 and H4 acetylation within genomic regions surrounding the GRE. ChIP experiments showed that p300 was recruited to the DUSP1 GRE, and RNA interference experiments demonstrated that reduction of p300 decreased glucocorticoid-stimulated DUSP1 gene expression and histone H3 hyperacetylation. Furthermore, overexpression of p300 potentiated glucocorticoid-stimulated activity of a reporter gene containing the DUSP1 GRE, and this coactivation effect was compromised when the histone acetyltransferase domain was mutated. ChIP-reChIP experiments using GR followed by p300 antibodies showed significant enrichment of the DUSP1 GRE upon glucocorticoid treatment, suggesting that GR and p300 are in the same protein complex recruited to the DUSP1 GRE.

**Conclusions/Significance:**

Our studies identified a functional GRE for the DUSP1 gene. Moreover, the transcriptional activation of DUSP1 by glucocorticoids requires p300 and a rapid modification of the chromatin structure surrounding the GRE. Overall, understanding the mechanism of glucocorticoid-induced DUSP1 gene transcription could provide insights into therapeutic approaches against inflammatory diseases.

## Introduction

Glucocorticoids are steroid hormones that exhibit potent anti-inflammatory effects through two main mechanisms. First, they inhibit the transcription of proinflammatory genes, such as cytokines, chemokines, and adhesion molecules via suppression of the transcriptional activation induced by AP-1 and NFκB [1], [2], [3], [4], [5]. Second, they induce genes that antagonize the inflammatory response, including the glucocorticoid-induced leucine zipper (GILZ) and dual specificity phosphatase-1 (DUSP1, a.k.a. mitogen-activated protein kinase phosphatase-1, MKP-1, Entrez GeneID: 1843) [6].

DUSP1 opposes the inflammatory response by blocking crucial signaling pathways. DUSP1 is a member of a large family of multifunctional phosphatases that resides in the nucleus and specifically dephosphorylates and inactivates members of the MAPK family, such as JNK, p38 MAPK, and ERK [7], [8]. These MAPKs play important roles in the stimulation of the inflammatory response by increasing the expression of many proinflammatory mediators [9], [10]. For example, cytokines, such as tumor necrosis factor α (TNF-α) and interleukin-1β (IL-1β), and endotoxins, such as lipopolysaccharides, have been shown to activate p38 MAPK, which in turn phosphorylates and activates downstream MAPKAP kinase 2 (MK2) [11], [12]. MK2 then phosphorylates and inactivates ZFP36 (also known as tristetraprolin, TTP). ZFP36 destabilizes the mRNA of many proinflammatory genes by binding to an AU-rich element (ARE) located at the 3′ untranslated region (UTR) of their mRNA [11], [12]. Increased expression of DUSP1 attenuates p38 MAPK signaling, which disrupts the signal leading to the induction of pro-inflammatory gene expression.

Mouse knockout studies support this role. Macrophages isolated from mice lacking DUSP1 gene (*Dusp1* ^−/−^) have prolonged activation of JNK and p38 MAPK, resulting in overexpression of inflammatory genes [13], [14]. Similarly, in LPS-induced endotoxemia and collagen-induced arthritis (experimental models of acute or chronic inflammation, respectively), *Dusp1* ^−/−^ mice show exaggerated inflammatory responses [15]. Lastly, in *Dusp1* ^−/−^ macrophages, the ability of glucocorticoids to inhibit the expression of specific pro-inflammatory genes is significantly compromised [15]. Overall, studies of various inflammatory disease models with *Dusp1* ^−/−^ animals support the critical role of DUSP1 in regulating anti-inflammatory responses mediated by glucocorticoids [16], [17], [18].

Despite the importance of DUSP1 in glucocorticoid-regulated inflammatory responses, how glucocorticoids regulate its expression is poorly understood [6], [19]. Glucocorticoids act through the intracellular glucocorticoid receptor (GR) to exert their biological functions. GR is a transcriptional regulator, which, upon binding to cognate ligands, occupies specific genomic regions called glucocorticoid response elements (GREs) [20]. These GREs are typically composite elements, composed of multiple cis-acting elements that work in a combinatorial fashion to direct a complete glucocorticoid response [21], [22]. Among these cis-acting elements are often multiple GR binding sites as well as binding sites for other transcriptional regulators. As each of these GREs appear to be different, it is likely that compositionally and/or conformationally distinct transcriptional regulatory complexes assemble at each locus, resulting in distinct mechanisms of transcriptional regulation at each GR target gene. As DUSP1 plays an important role in mediating the anti-inflammatory response of glucocorticoids, dissecting the mechanisms by which GR activates DUSP1 gene transcription is critical for our fundamental understanding of anti-inflammatory action exhibited by glucocorticoids. In this report, we identified a GRE for the human DUSP1 gene. We also analyzed the effect of glucocorticoids on the chromatin structure and acetylation status of histones surrounding the GRE. Finally, using RNA interference and chromatin immunoprecipitation approaches, we identified a transcriptional cofactor participating in GR-activated DUSP1 gene transcription.

## Methods

### Cell Culture

A549 cells (type II alveolar lung epithelium cells; ATCC CCL85) [23] were cultured in DMEM (Cellgro) with 5% fetal bovine serum (FBS; J R Scientific). When cells were treated with dexamethasone (DEX, a synthetic glucocorticoid, Sigma) or DMSO (vehicle control), DMEM with 5% charcoal stripped FBS (J R Scientific) was used.

### ChIP

A549 cells were grown to confluence and treated with 0.5 µM DEX or DMSO, then cross-linked by using formaldehyde at a final concentration of 1% at room temperature for 5 minutes. The ChIP protocols were otherwise as previously described [24], [25].

### ChIP-reChIP

A549 cells were grown to confluence (4×150 mm plates per treatment) and treated with 0.5 µM DEX or DMSO, then cross-linked by using formaldehyde at a final concentration of 1% at room temperature for 5 minutes. Reactions were quenched with 0.125 M glycine for 5 min. Cells were washed with PBS, scraped in cell lysis buffer (50 mM HEPES-KOH at pH 7.4, 1 mM EDTA, 150 mM NaCl, 10% glycerol, 0.5% Triton X-100) supplemented with protease inhibitor cocktails (Calbiochem), and incubated for 10 min at 4°C. Crude nuclei were collected by centrifugation at 600 x g for 5 min at 4°C then resuspended in 1.5 ml of ice-cold RIPA buffer (10 mM Tris-HCL at pH 8.0, 1 mM EDTA, 150 mM NaCl, 5% glycerol, 1% Triton X-100, 0.1% sodium deoxycholate, 0.1% SDS, supplemented with protease inhibitor). Chromatin was fragmented with 18 pulses of 20 sec at 30% power with a Branson Sonifier 250 sonicator at 4°C. To remove insoluble components, samples were centrifuged at 13,000 rpm for 15 min at 4°C, and the supernatant was recovered.

For the first round of IP, two aliquots from each treatment were taken. 8 µg of rabbit polyclonal GR (N499) antibody was used to immunoprecipitate GR-bound chromatin or 8 µg of rabbit polyclonal IgG was used as a control for each aliquot, per treatment. The samples rotated overnight at 4°C. 120 µl of 50% protein A/G plus-agarose bead slurry (Santa Cruz Biotechnology) was then added to each immunoprecipitation and rotated for 2 h at 4°C. After incubation, beads were washed with 1 ml of solution per wash as follows: twice with RIPA, twice with RIPA containing 510 mM NaCl, twice with LiCl buffer (20 mM Tris at pH 8.0, 1 mM EDTA, 250 mM LiCl, 0.5% NP-40, 0.5% sodiumdeoxycholate), and twice with TE. Between each wash, samples were rotated at room temperature for 5 min, followed by 1 min centrifugation at 5,000 rpm. All washes were supplemented with protease inhibitors. Immmunocomplexes were eluted by adding 75 µl TE with 10 mM DTT and incubated at 37°C for 30 min. The samples were centrifuged at 800 x g for 2 minutes at room temperature, and the supernatant was transferred to a clean 1.5 ml tube. An additional 14 µl of RIPA was added to dilute the sample.

For the second round of IP, 8 µg of p300 (rabbit polyclonal, Santa Cruz Biotechnology) was used in all samples and rotated overnight at 4°C. Again, we added 120 µl of 50% protein A/G plus-agarose bead slurry and washed as described, modified only by replacing the final two TE washes with one wash in RIPA buffer. After removing the last wash buffer, 75 µl of proteinase K solution (TE pH 8.0, 0.7% SDS, 200 µg/ml proteinase K) was added to each IP. Reactions were incubated for 3 h at 55°C, then overnight at 65°C to reverse formaldehyde cross-links. DNA was purified using a QIAquick PCR purification kit (Qiagen) and eluted in 60 µl of Elution Buffer (Qiagen).

### Transfection

To transfect A549 cells, we used Lipofectamine 2000 (Invitrogen) according to the technical manual in the 24-well format. Twenty-four hours post-transfection, cells were treated with either DMSO or 0.5 µM DEX for 18–24 h. Cells were then harvested, and luciferase activity was measured by a Dual-Luciferase Reporter Assay kit (Promega) according to the technical manual. pCI-p300 wild type and pCI-p300ΔHAT were provided by Mike Stallcup (USC) [26].

### Cloning and Site Directed Mutagenesis

The human DUSP1 genomic region containing the predicted GR binding site was amplified by PCR using specific primers. The PCR fragment was restriction-digested and subcloned into the pGL4-TATA reporter. A mutagenesis kit (QuikChange) was used to make site-directed mutations per the manufacturer's instructions (Stratagene).

### DNase Hypersensitivity Assay

A549 cells were grown to confluence in 6-well plates then treated with DMSO or 0.5 µM DEX for 10 min. Cells were washed then scraped in 1 ml ice cold DNase I Buffer + NP40 (20 mM HEPES pH 7.4, 0.5 mM CaCl_2_, 5% glycerol, 3 mM MgCl_2_, 0.2 mM spermine, 0.2 mM spermidine, 0.2% NP40). Cells were vortexed for 10 seconds to homogenize, then centrifuged at 500 x g in a microfuge for 5 minutes at 4°C. Pellets were resuspended in 100 µl DNase I Buffer without NP40, then equilibrated to room temperature for 10 min. Samples were digested with 5 units of DNase I (QIAGEN) for 5 minutes at 37°C, and reactions were stopped by addition of equal volume (100 µl) 2× Stop Reaction Buffer (50 mM Tris-HCl pH 8.0, 1% SDS, 200 mM NaCl, 12.5 mM EDTA) with 200 µg/ml Proteinase K, and incubated at 65°C for 4 hours or overnight. DNA was purified using a PCR purification kit (QIAGEN), and samples were measured and diluted to use 50 ng/well for qPCR analysis.

### RNA interference (RNAi)

p300 (QIAGEN), CBP (Santa Cruz Biotechnology), and a scramble sequence (control, QIAGEN) were reverse transfected with HiPerFect transfection reagent (QIAGEN) according to the technical manual. For gene expression analyses, reverse transfection was done in a 24-well plate. Forty-eight hours post-transfection, cells were treated with either DMSO or 0.5 µM DEX for 5 hours, and total RNA was then isolated. For western blot analyses, cells were collected from a 24-well plate 48 hours post-transfection. For ChIP experiments, reverse transfection was done in 10 cm plates. Again, 48 hours post-transfection, cells were treated with either DMSO or 0.5 µM DEX, this time for 10 minutes.

### Western Blot

A549 cells transfected with p300, CBP, or scramble (control) siRNA were collected 48 hours post-transfection in RIPA buffer (10 mM Tris-HCl pH 8.0, 1 mM EDTA, 150 mM NaCl, 5% glycerol, 0.1% sodium deoxycholate, 0.1% SDS, 1% Triton X-100, supplemented with protease inhibitors). Proteins were resolved and imaged as described [27. The following antibodies were used: p300 rabbit polyclonal IgG (sc-584; Santa Cruz Biotechnology), CBP mouse monoclonal IgG_1_ (sc-7300, Santa Cruz Biotechnology), β-Actin (C4) mouse monoclonal IgG_1_ (sc-47778; Santa Cruz Biotechnology), anti-rabbit IgG_1_-HRP (Cell Signaling), and anti-mouse IgG_1_-HRP (sc-2060; Santa Cruz Biotechnology).

### Micrococcal Nuclease (MNase)

A549 cells were grown to confluence and treated with DMSO or 0.5 µM DEX for 10 minutes. Cells were cross-linked with 1% formaldehyde for 3 minutes at 22°C, and reactions were quenched by adding glycine to a final concentration of 0.125 M. Cells were then rinsed in PBS and scraped in ice-cold MNase NP-40 Lysis Buffer (10 mM Tris pH 7.4, 10 mM NaCl, 3 mM MgCl_2_, 0.5% NP-40, 0.15 mM spermine, 0.5 mM spermidine). Cells were vortexed for 10 seconds to ensure thorough lysis before nuclei were collected by centrifugation at maximal speed in a microfuge for 5 minutes at 4°C. Nuclei were washed in ice-cold MNase Digestion Buffer without CaCl_2_ (10 mM Tris pH 7.4, 15 mM NaCl, 60 mM KCl, 0.15 mM spermine, 0.5 mM spermidine) and re-centrifuged. Pelleted nuclei were suspended in ice-cold MNase Digestion Buffer with CaCl_2_ (10 mM Tris pH 7.4, 15 mM NaCl, 60 mM KCl, 0.15 mM spermine, 0.5 mM spermidine, 1 mM CaCl_2_). Samples were treated with 1 unit of MNase (Nuclease micrococcal from Staphylococcus aureus, Sigma-Aldrich, N5386-200UN) for 10 minutes at 25°C. Reactions were stopped by addition of 80 µl MNase Digestion Buffer with CaCl_2_, 20 µl MNase Stop Buffer (100 mM EDTA, 10 mM EGTA), 75 µg Proteinase K, and 20 µl 10% SDS, and incubated at 65°C for 4 hours or overnight. Samples were then column purified using a PCR purification kit (QIAGEN). RNaseA (Millipore) was diluted to 0.1 µg/µl in TE, and 20 units were added to each sample for 2 hours at 37°C. Samples were then purified using a PCR purification kit (QIAGEN) and run on a 1.5% agarose gel. DNA fragments of 150 bp were purified using a gel extraction kit (QIAGEN).

Quantitative real-time PCR was used to measure the amount of distinct genomic fragments surrounding the DUSP1 GRE. For each primer set, a standard curve was generated using 1 µg, 0.25 µg, 0.0625 µg, and 0.015625 µg of human genomic DNA. The amount of a particular genomic fragment in the MNase sample was then calculated based on the standard curve.

### Nuclear Run-on

A549 cells were grown to confluence and treated with DMSO or 0.5 µM DEX for different times. Cells were then harvested and incubated in lysis buffer (10 mM Tris-HCl pH 7.4, 3 mM MgCl_2_, 10 mM NaCl, 150 mM sucrose and 0.5% NP40) at 4°C for 5 minutes. Nuclei were then isolated by centrifugation at 170 x g at 4°C for 5 min. The total nuclei from each of the DMSO or DEX treated samples were counted, and equal numbers of nuclei were used for *in vitro* transcription. The samples were split into two aliquots. One was incubated in 100 µl 2× *in vitro* transcription buffer (200 mM KCl, 20 mM Tris-HCl pH 8.0, 5 mM MgCl_2_, 4 mM dithiothreitol (DTT), 4 mM each of ATP, GTP and CTP, 200 mM sucrose and 20% glycerol) plus 8 µl biotin-UTP (Roche), and the other in 100 µl 2× *in vitro* transcription buffer plus 8 µl UTP (negative control) for 30 minutes at 29°C. 6 µl of 250 mM CaCl_2_ and 6 µl of RNAse free DNase (Roche) (10 U/µl) were then added to stop the reactions. Total RNA was then isolated using Nucleospin RNA II (Macherey-Nagel).

Dyna beads M-280 (Invitrogen) were washed twice in solution A (0.1 mM NaOH, 0.5 M NaCl) for 5 min, once in solution B (0.1 M NaCl) for 5 min, and then resuspended in binding/wash buffer (10 mM Tris-HCl pH 7.5, 1 mM EDTA and 2 M NaCl) plus 1 µl (40 U) RNasin per 100 µl of beads. 50 µl of beads (in binding/wash buffer) were then added to RNA, incubated at 42°C for 20 min, and then shaken for 2 h at room temperature. Afterward, the beads were isolated and the supernatant discarded. The beads were then washed once (5 min) with 500 µl 15% formamide plus 2× saline-sodium citrate (SSC) buffer, twice with 1 ml 2× SSC buffer, then resuspended in 30 µl RNase and DNase free water. 10 µl of beads were used for each reverse transcription (RT) reaction prior to qPCR.

### RNA, RT, qPCR

Total RNA was isolated from cells by using QIAshredder and RNeasy kits (Qiagen) or a NucleoSpin RNAII kit (Macherey-Nagel). To synthesize random-primed cDNA, 0.5 µg of total RNA (10 µl), 4 µl of 2.5 mM dNTP, and 2 µl of random primers (New England Biolabs) were mixed and incubated at 70°C for 10 min. A 4-µl cocktail containing 25 U of Moloney Murine Leukemia Virus (M-MuLV) Reverse Transcriptase (New England Biolabs), 10 units of RNasin (Promega), and 2 µl of 10× reaction buffer (New England Biolabs) was then added. The reaction was incubated at 42°C for 1 h, then 95°C for 5 min.

The resultant cDNA was diluted to 200 µl with water, and 2.5 µl was used to perform qPCR using EVA QPCR SuperMix Kit (Biochain) per the manufacturer's protocol. qPCR was performed in either a 7900 HT, 7500 HT, or StepOne PCR System (Applied Biosystems) and analyzed by using the ▵▵-Ct method as supplied by the manufacturer (Applied Biosystems). *Rpl19* expression was used for internal normalization. All primer sequences are presented in Table S1.

## Results

### Induction of DUSP1 gene transcription by glucocorticoids in human A549 cells

**Figure 1:**
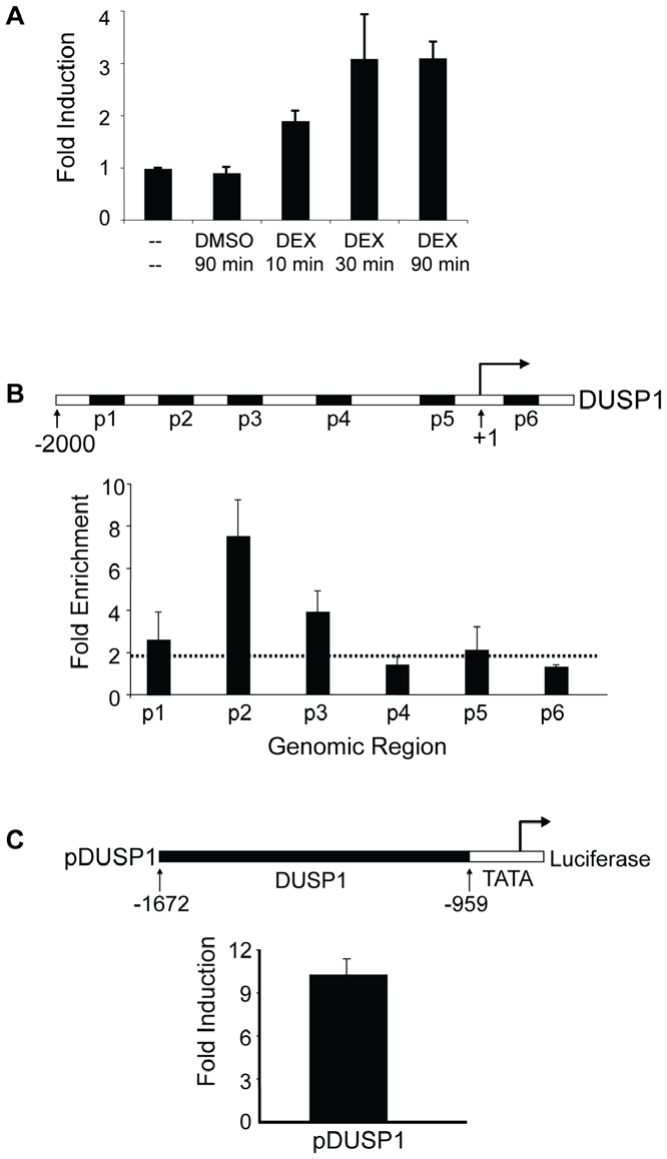
Identification of DNA elements mediating glucocorticoid response in the human DUSP1 gene. **A** A549 cells were untreated, treated with DMSO for 90 min, or treated with DEX (0.5 µM) for 10 min, 30 min, and 90 min as shown. Nuclei were used for nuclear run-on experiments with biotin-UTP to monitor *in vitro* transcription. Newly synthesized RNA was isolated and used to make cDNA. qPCR was performed to monitor changes in transcription rates using primers specific to *Dusp1* and *Rpl19* (control). Data represent the standard error mean (SEM) of the fold induction (DMSO- or DEX-treated cells divided by untreated cells) from at least three experiments. **B** Identification of GR binding regions in human DUSP1 using ChIP scanning across the first 2 kb of the human DUSP1 promoter. A549 cells were treated with DMSO or DEX (0.5 µM) for 10 min, and ChIP was performed with GR-specific antibody as described. DUSP1 gene schematic shows the location of six primers used (black boxes labeled p1-p6) for qPCR analyses. The regions of the DUSP1 gene (relative to the TSS) amplified by these primers are: p1 (−1815 to −1717), p2 (−1421 to −1333), p3 (−1191 to −1118), p4 (−662 to −543), p5 (−160 to −54), and p6 (+98 to +215). Data represent the SEM of the fold enrichment (DEX-treated cells divided by DMSO-treated cells) from at least three experiments. **C** DUSP1 GR binding region mediates glucocorticoid response. −1672 to −959 (relative to the TSS) of the human DUSP1 genomic region was subcloned into pGL4-TATA reporter plasmid to create pDUSP1. pDUSP1 was transfected into A549 cells along with an expression vector for human GR. 24 h post-transfection, cells were treated with DMSO or DEX (0.5 µM) for 18–20 h. Luciferase assay data represents the SEM of the fold induction of luciferase activity (DEX-treated cells divided by DMSO-treated cells) from at least three experiments.

Previous studies have shown that DUSP1 gene expression is induced by glucocorticoids in A549 cells [28]. To confirm that the DUSP1 gene is a direct target of GR and to determine the kinetics of glucocorticoid-induced transcription, we performed nuclear run-on experiments. We isolated nuclei from A549 cells that were treated with the synthetic glucocorticoid dexamethasone (DEX) for 10, 30, and 90 minutes. Nascent transcripts from these nuclei were labeled with biotin-UTP, and then isolated using magnetic beads. After random-primed cDNA was synthesized, qPCR was performed to measure the change of transcript levels using DUSP1 specific primers. As shown in Fig. 1A, transcription of DUSP1 is induced rapidly, increasing approximately two fold after just 10 minutes of DEX treatment (compared to 90 minutes DMSO-treated samples and no treatment), with levels continuing to increase after 30 and 90 minutes (Fig. 1A). These results demonstrate that glucocorticoids rapidly initiate transcription of DUSP1, and that it is likely directly regulated by GR.

### Identification of GREs in the human DUSP1 gene

As our data revealed that glucocorticoids stimulate DUSP1 through transcriptional initiation, we wished to further probe the mechanism by which GR induces DUSP1 transcription. To this end, we used a chromatin immunoprecipitation (ChIP) scanning approach [23] to systematically locate the GR binding regions in the vicinity of the human DUSP1 transcription start site (TSS). A549 cells were treated with DMSO or DEX for 10 minutes, when we first observed transcriptional initiation, then subjected to a standard ChIP procedure [25] of formaldehyde cross-linking of A549 cells, shearing of chromatin to approximately 200–500 bp fragments, and precipitating cross-linked GR:DNA complexes with GR-specific antibody. IgG antibody was used as a negative control for all ChIP experiments. We performed qPCR on the precipitated DNA fragments using six pairs of oligonucleotide primers that amplify approximately 100 bp segments (Fig. 1B), spaced at 300–500 bp intervals. As the average size of the sheared DNA fragments is 300–500 bp, this arrangement of amplified sequences can scan effectively for GR occupancy in the first 2 kb upstream of the DUSP1 TSS. We found that two primer pairs, flanking −1421 to −1333 and −1191 to −1118 respectively, exhibited significant enrichment in GR ChIP (Fig. 1B), but not with IgG alone (data not shown). These results reveal that GR binding localizes between −1421 and −1118 of the TSS.

We next tested whether this GR binding region was a mediator of glucocorticoid response. We subcloned the genomic region extending approximately 200–300 bp upstream and downstream of our ChIP fragments (−1672 to −959) into an E4 TATA box containing pGL4 luciferase reporter to test whether the identified GR-binding regions are glucocorticoid-responsive (Fig. 1C). We transfected this reporter plasmid (pDUSP1) along with an expression vector encoding human GR cDNA into A549 cells. After 24 hours, cells were treated with DMSO or DEX for 18 to 20 hours. Cells were then lysed, and firefly luciferase assay was performed. We found that firefly luciferase activity in DEX-treated cell lysates was significantly higher than that of DMSO-treated cell lysates (Fig. 1C). These results indicate that the −1672 to −959 region of the DUSP1 gene promoter is a functional GRE.

**Figure 2:**
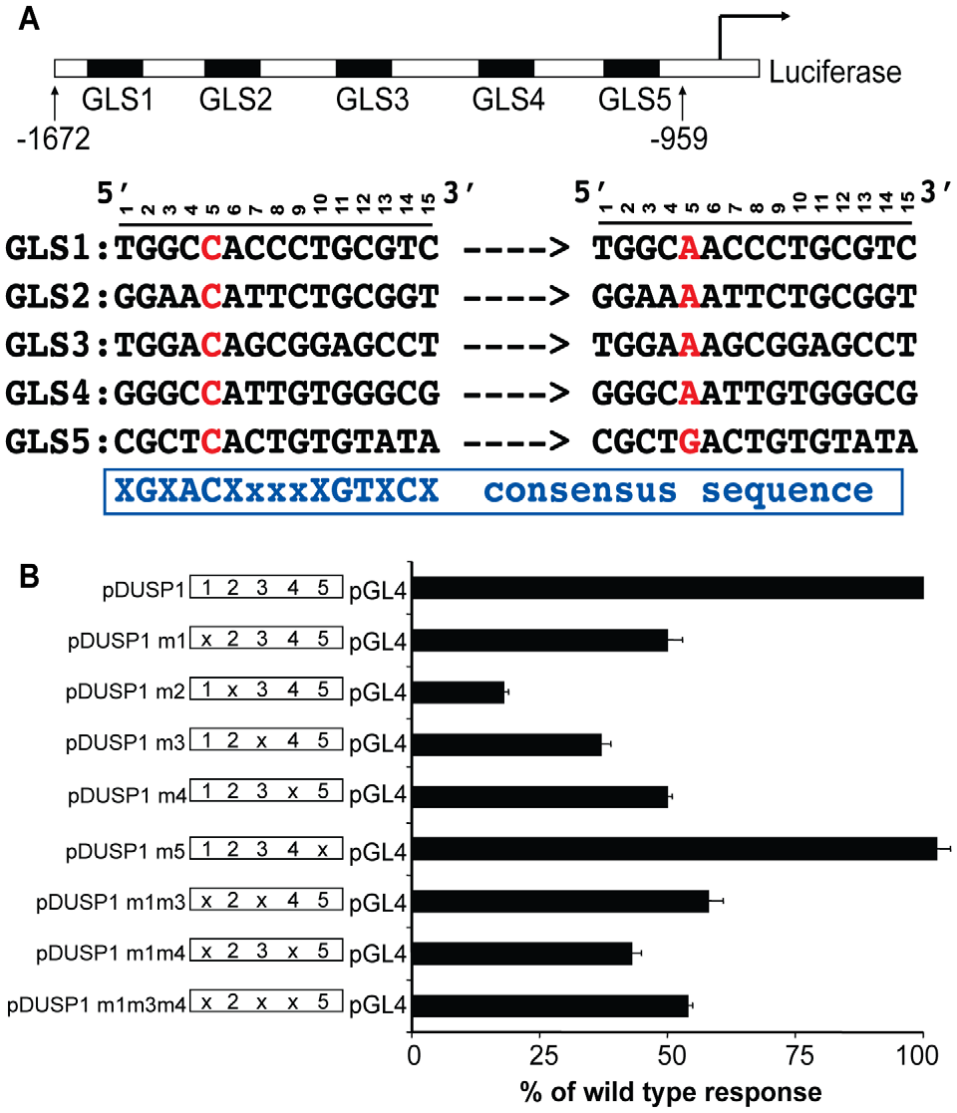
Mutational analyses of GRE-like sequences (GLS) in the DUSP1 gene. **A** Five GLSs identified in the −1672 to −959 region of the human DUSP1 promoter. The locations of five GRE like sequences are: GLS1 (−1382 to −1368), GLS2 (−1337 to −1323), GLS3 (−1254 to −1240), GLS4 (−1154 to −1140), GLS5 (−1030 to −1018). The position of each nucleotide in each GLS is indicated. The specific point mutations made in each GLS are shown in red. In GLS1-GLS4, position 5 was mutated from C to A. In GLS5, position 5 was mutated from C to G. The consensus GR binding sequence is indicated in blue. **B** Wild type and mutant reporter plasmids were transfected into A549 cells along with a human GR expression vector. 24 h post-transfection, cells were treated with DMSO or DEX (0.5 µM) for 18–20 h. Luciferase assay data represent the SEM of the fold induction of luciferase activity (DEX-treated cells divided by DMSO-treated cells) from at least three experiments. Results are shown as a percent of wild type DUSP1 response. For mutant plasmids, the mutation at distinct GLSs is indicated (e.g. m1 indicates a mutation at GLS1).

To further resolve binding in this region, we looked for direct GR binding sites by searching for a consensus GR binding sequence. Based on a compilation of known GR binding sequences (S. Cooper and K. Yamamoto, unpublished data), we searched for motifs matching the loose consensus sequence XGXACXxxxXGTXCX. We identified a 15 bp sequence that closely matched this consensus GRE in the −1672 to −959 region of the human DUSP1 promoter (GLS2). The search yielded four more degenerate potential binding sites (GLS 1, 3, 4, 5, Fig. 2A). To gauge the importance of each GLS to GRE activity, we compared the wild type sequence to ones mutated at each of the GLSs at position 5, where GR makes direct contact and is critical for specific binding [29]. We transfected these mutant constructs into A549 cells and found that mutating GLS2 (pDUSP1m2) severely reduced the glucocorticoid response, whereas a mutation at GLS5 (pDUSP1m5) had no effect (Fig. 2B). Mutations at GLS1, GLS3, and GLS4 (pDUSP1m1, pDUSP1m3, pDUSP1m4) showed a modest reduction of glucocorticoid response (Fig. 2B). To verify the contribution of these sites, we performed double mutations of GLS1 and GLS3 (pDUSP1m1m3), GLS1 and GLS4 (pDUSP1m1m4), and GLS3 and GLS4 (pDUSP1m3m4). These double mutations did not further compromise DEX response, nor did the triple mutant (GLS1, GLS3 and GLS4, pDUSP1m1m3m4). In all cases, the response of multiple mutations to DEX was similar to single mutations within GLS1, GLS3, and GLS4 (Fig. 2B). Overall, this mutational analysis indicates that GLS2, which best fits the canonical GRE consensus, is essential for conferring glucocorticoid response, whereas GLSs 1, 3, and 4, which are more divergent, may play accessory roles that could contribute to hormone response under some conditions.

### GRE containing GLS2 has active response element chromatin marks

To further understand the mechanisms underlying glucocorticoid-regulated DUSP1 gene transcription, we investigated the effects of glucocorticoids on the chromatin structure of the DUSP1 gene. Chromatin architecture plays a critical role in the control of eukaryotic gene transcription *in vivo* because it determines the accessibility of genomic regulatory sequences to the transcriptional machinery. Based on their sensitivity to cleavage when intact nuclei are exposed to DNA-modifying agents, such as endonuclease DNase I, we can analyze the accessibility of the chromatin structure of specific genomic regions.

**Figure 3:**
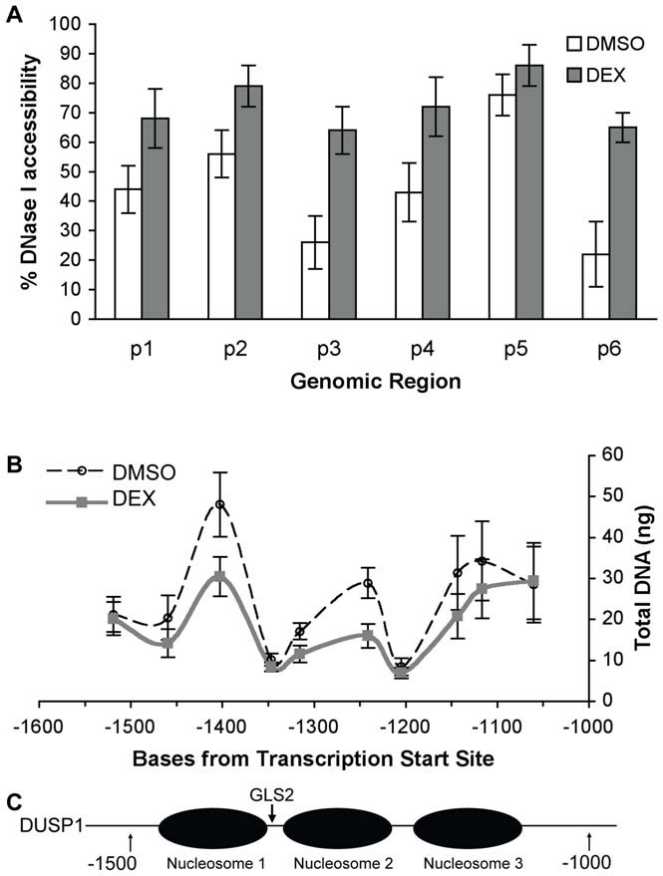
DUSP1 DNaseI accessibility and nucleosome mapping in response to glucocorticoids. **A** DNase I accessibility assay was performed with A549 cells treated for 10 minutes with DMSO or DEX (0.5 µM). DNase I digestion lasted 5 min, and fragments were analyzed by qPCR using the same primers (p1-p6) used for ChIP qPCR in Figure 1B. 50 ng DNA was used per well for qPCR. Results are shown as a percent increase of DNase I accessibility relative to DNA not digested by DNase I, where 100% accessibility represents a complete digestion of DNA. Data represent the SEM of percentage increase (DNaseI-cut cells divided by uncut cells) from at least four experiments. **B** Nucleosome positioning was analyzed with micrococcal nuclease (MNase) digestion for the −1550 to −1050 region of the DUSP1 promoter. A549 cells were treated for 10 minutes with DMSO or DEX (0.5 µM) before digestion with MNase for 10 min. qPCR was performed with primers annealing approximately every 50 bp. Results are shown as the amount of each genomic fragment in the MNase sample, calculated based on a standard curve from genomic DNA titrations with each primer set. **C** Three potential nucleosomal regions are located at approximately −1500 to −1350 (nucleosome 1), −1350 to −1200 (nucleosome 2), and −1200 to −1050 (nucleosome 3) of the human DUSP1 gene. GLS2 is located between nuclesomes 1 and 2.

We first tested whether glucocorticoids affect DNase I accessibility within the DUSP1 promoter. Nuclei from A549 cells treated with DMSO or DEX for 10 minutes were subject to cleavage by DNase I for 5 minutes. DNA fragments were then isolated and analyzed with qPCR using primers from the previously described ChIP experiments. We found that the region from −2000 to +1 (TSS) of DUSP1 was already sensitive to DNase I before DEX treatment (compared to DNA not digested by DNase I, Fig. 3A). In contrast, the promoter region of α-fetoprotein, a liver-specific gene not expressed in A549 cells, was sensitive neither to DNase I digestion nor DEX treatment (Fig. S1). With DUSP1, DEX treatment markedly increased DNase I accessibility in all areas examined except −160 to −64 (Fig. 3A), which was already very sensitive to DNase I prior to DEX treatment (Fig. 3A). These results indicate that DEX treatment and GR binding can further “open up” the chromatin structure of most genomic regions surrounding the DUSP1 promoter.

This opening of chromatin within the GRE suggests that nucleosomes have been repositioned or remodeled. Glucocorticoids have previously been shown to induce chromatin remodeling; thus, they must either disrupt a nucleosome assembly or change the position of nucleosomes in the genome (41). To test this, we used MNase to map the position of nucleosomes surrounding the DUSP1 gene. We treated A549 cells with DMSO or DEX for 10 min. Nuclei were isolated, cross-linked with formaldehyde, and digested with MNase. The MNase sensitivity of the chromatin was then analyzed using qPCR with primers designed to anneal to the DNA approximately every 50 bp from −1550 to −1050 of the DUSP1 TSS. Because the nucleosomal regions are less accessible to MNase, we recovered more DNA fragments from these regions. In contrast, the recovery of DNA fragments from linker regions was lower, as most of these fragments were digested by MNase. In untreated cells, three solid nucleosome positions are evident: from −1500 to −1350 (nucleosome 1), −1350 to −1200 (nucleosome 2), and −1200 to −1050 (nucleosome 3) (Fig. 3B). Consistent with our DNase sensitivity assay, addition of DEX appeared to increase the sensitivity to MNase in nucleosomes 1 and 2 (Fig. 3B). These data indicate that a glucocorticoid-dependent remodeling of chromatin structure within the DUSP1 GRE is associated with gene activation.

### Glucocorticoids increase histone H3 and H4 acetylation of the DUSP1 gene promoter

**Figure 4:**
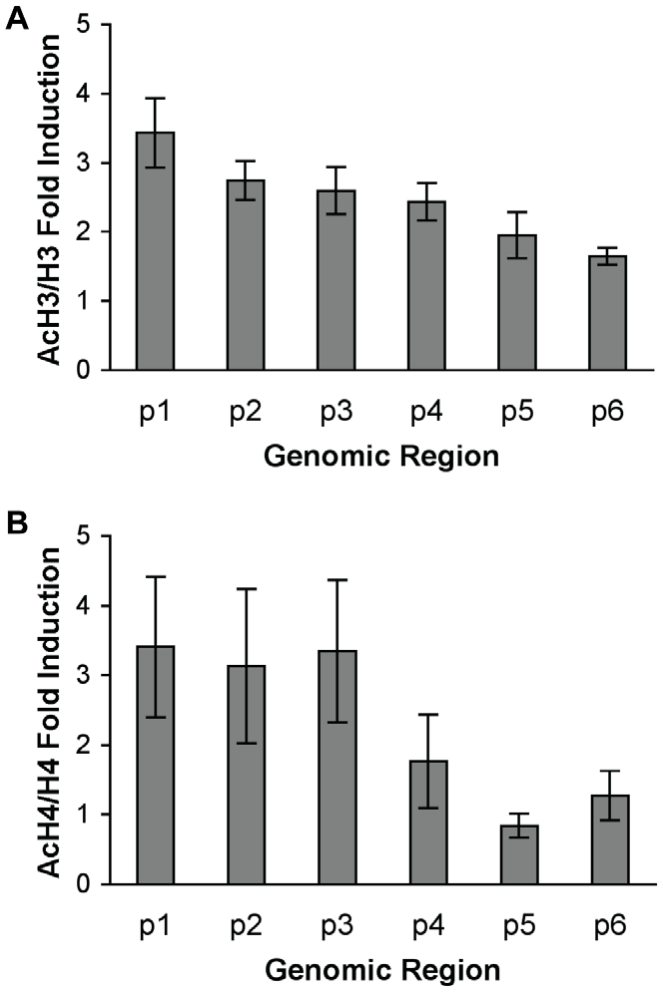
Glucocorticoids increased histone H3 and H4 acetylation of the DUSP1 gene promoter. **A** A549 cells were treated with DMSO or DEX (0.5 µM) for 10 min, and ChIPs were performed with antibodies against both total histone H3 and multiple acetylated lysines on histone H3 (AcH3). qPCR was performed with the same six primers used for ChIP qPCR in Figure 1B. AcH3 data was normalized to total H3 values and represents the SEM of the fold induction (DEX-treated cells divided by DMSO-treated cells) from at least four experiments. **B** Identical experiments and calculations as in **A**, with ChIP antibodies against both total histone H4 and multiple acetylated lysines on histone H4 (AcH4).

An increase in the acetylation of histones H3 and H4 has been correlated with both gene transcription and active DNA control elements such as response elements and enhancers [30]. We treated A549 cells for 10 minutes with DMSO or DEX, then performed ChIP experiments using antibodies that recognize multiple acetyl lysines in the tails of either histone H3 (AcH3) or H4 (AcH4) to examine whether glucocorticoids affect the overall histone acetylation status of the DUSP1 gene promoter. Total H3 and H4 levels were also monitored by ChIP, as the levels of AcH3 and AcH4 are associated with the overall density of histone in each genomic region. Thus, the ratio of AcH3/H3 or AcH4/H4 accurately reflects the change of histone acetylation. In comparison to ChIP done with control IgG antibodies, we found that AcH3/H3 and AcH4/H4 levels were already enriched before DEX treatment in all genomic regions tested (Fig. S2). These results indicate that the −2000 to +1 region of the DUSP1 gene was already acetylated before DEX treatment. DEX treatment then further increased the AcH3/H3 levels in all regions examined (Fig. S2A, and DEX/DMSO fold induction in Fig. 4A). Conversely, DEX treatment potentiated AcH4/H4 levels in the genomic region from −1815 to −543, but had no effect in the region from −160 to +215 (Fig. S2B and Fig. 4B).

**Figure 5:**
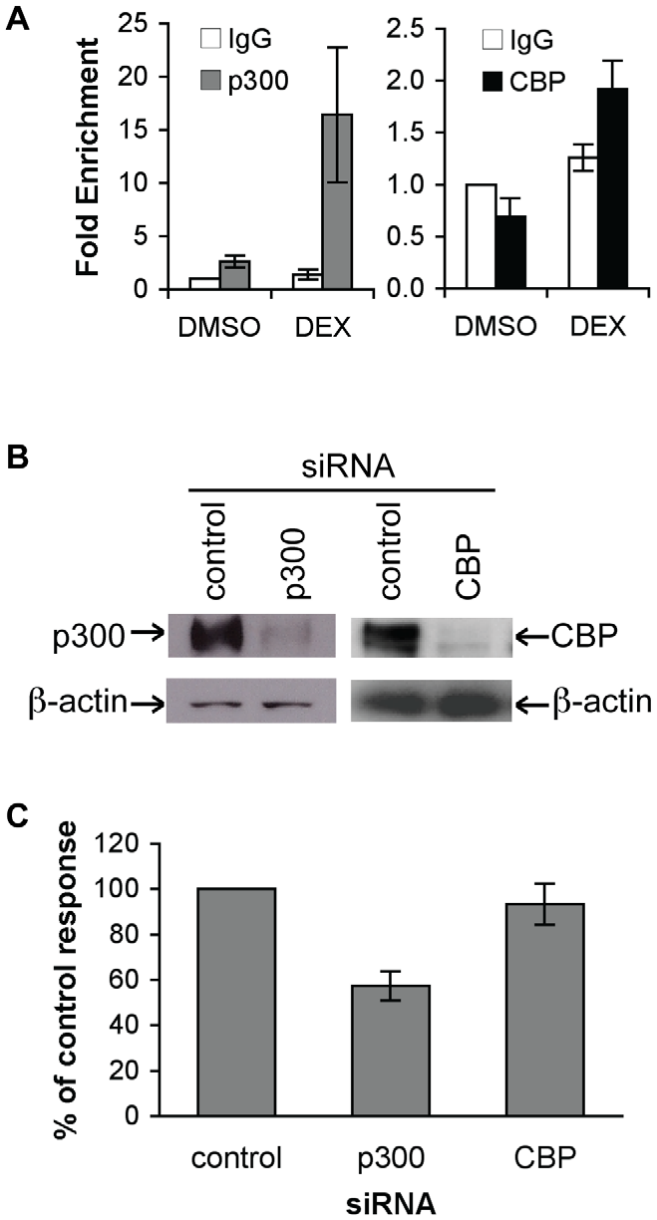
p300 is a transcriptional coactivator for GR-regulated DUSP1 gene transcription. **A** Glucocorticoid treatment increased the level of p300 and CBP at the DUSP1 GLS2 region. A549 cells were treated with DMSO or DEX (0.5 µM) for 10 min, and ChIP was performed with antibodies against p300, CBP and IgG (control). Data is shown from qPCR analyses with primers specific to the DUSP1 GLS2. **B** RNAi knockdown causes substantial reduction of p300 protein and CBP protein. A549 cells reverse transfected with p300, CBP or scramble siRNA were harvested 48 h post-transfection, then probed by Western blot using an antibody against p300 or CBP, with β-actin as a loading control. **C** RNAi against p300 but not CBP decreased DEX-induced DUSP1 gene expression. p300, CBP or scramble (control) siRNA was transfected into A549 cells. 48 h post-transfection, cells were treated with DMSO or DEX (0.5 µM) for 5 h. Total RNA was isolated and converted to cDNA. qPCR was used to monitor the expression of DUSP1 gene. The expression of Rpl19 gene was used as an internal control. Data represents the SEM of DEX-induced DUSP1 gene expression (DEX-treated cells divided by DMSO-treated cells), as a percent of the control siRNA response from at least three experiments.

Next, we wished to determine the histone acetyltransferase (HAT) that acetylates H3 and H4 at the DUSP1 gene. Several coactivators for GR, including GCN5, PCAF, CBP/p300, have been shown to contain histone acetyltransferase (HAT) activity. We reason that if a coactivator is directly involved in glucocorticoid-activated DUSP1 gene transcription, it should be present on the DUSP1 promoter upon DEX treatment. To determine which of these factors are directly involved in glucocorticoid-activated DUSP1 gene transcription, we treated A549 cells with DMSO or DEX for 10 minutes, then ChIPed for GCN5, PCAF, CBP and p300 in the areas surrounding the DUSP1 GRE. Of these, p300 was present before treatment with glucocorticoids (relative to ChIP with IgG control), with levels increasing approximately 16 fold after only 10 minutes of DEX treatment (Fig. 5A). In contrast, CBP was absent without treatment, but was enriched approximately 1.9 fold with 10 minutes of DEX treatment (Fig. 5A). We used RNA interference (RNAi) to reduce the expression of p300 and CBP in A549 cells (Fig. 5B), then tested whether this compromised the ability of DEX to stimulate the expression of the DUSP1 gene (Fig. 5C). Compared to cells transfected with negative control siRNA, p300 knockdown resulted in approximately a two-fold increase of basal expression of DUSP1 gene (Fig. S3). However, the ability of DEX to induce DUSP1 gene expression was decreased approximately 40% (Fig. 5C). These results indicate that under basal conditions, p300 inhibits DUSP1 gene expression, while upon DEX stimulation it contributes to induction of DUSP1 gene expression. CBP RNAi affected neither basal nor DEX-stimulated expression of DUSP1 gene (Fig. S3 and Fig. 5C).

### p300 is involved in glucocorticoid-induced hyperacetylation of H3 in genomic regions surrounding GRE

**Figure 6:**
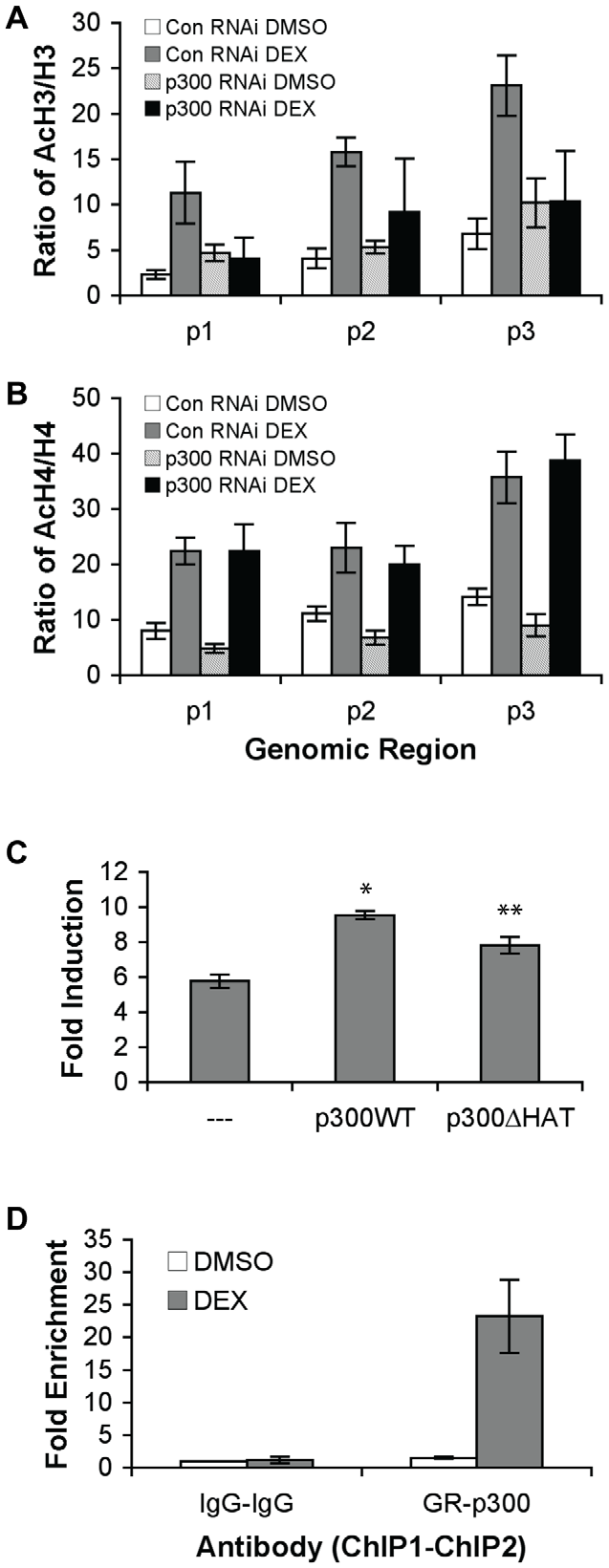
p300 contributes to hyperacetylation of H3 but not H4 in DUSP1 GRE-surrounding region. **A** A549 cells reverse transfected with p300 or scramble (control) RNAi were treated 48 h post-transfection with DMSO or DEX (0.5 µM) for 10 min. ChIPs were performed for AcH3 and H3 as described in Figure 4A, and **B** for AcH4 and H4 as described in Figure 4B. RNAi knockdown of p300 decreases DEX-induced hyperacetylation of H3 but not H4. Data represent the SEM of fold change relative to IgG ChIP from at least four experiments. **C** pCI, pCI-p300 wild type (WT), or pCI-p300ΔHAT mutant was co-transfected with pDUSP1 along with an expression vector for human GR into A549 cells. Cells were treated with DMSO or DEX as described in Figure 1C. Luciferase assay data represents the SEM of the fold induction of luciferase activity (DEX-treated cells divided by DMSO-treated cells) from at least five experiments. *p300WT caused significant induction from pCI control (student t-test, p<0.0001). ** p300ΔHAT mutation caused significant reduction from p300WT response (student t-test, p = 0.01). **D** ChIP-reChIP was performe with GR antibody followed by p300 antibody to isolate fragments bound to both GR and p300. Fragments were analyzed by qPCR with primers corresponding to GLS2. Data represent the SEM of the fold enrichment (DMSO- or DEX-treated cells divided by DMSO-treated IgG-ChIP cells) from at least three experiments.

We used RNAi to decrease the expression of p300 to further investigate its role in DEX-induced histone hyperacetylation. As shown in Fig. 6A, the levels of AcH3/H3 in p300 RNAi cells were similar to those of control RNAi cells before DEX treatment. However, while a 10 minute DEX treatment significantly induced AcH3/H3 levels in control RNAi cells, the ability of DEX to increase AcH3/H3 levels was markedly compromised in p300 knockdown cells (Fig. 6A). These results suggest that p300 is involved in DEX-induced histone H3 hyperacetylation surrounding the DUSP1 GRE, though it does not participate in acetylation of H3 before DEX treatment. Interestingly, AcH4/H4 levels in p300 RNAi cells were similar to those of control RNAi cells before and after 10 min DEX treatment (Fig. 6B). Thus, decreasing p300 expression does not affect DEX-induced histone H4 hyperacetylation.

To further confirm the importance of p300-induced histone acetylation in DEX-induced DUSP1 gene transcription, we compared the ability of wild type (WT) p300 and a p300 mutant with reduced histone acetyltransferase (HAT) activity (p300ΔHAT) to coactivate DEX-induced pDUSP1 reporter gene expression (Fig. 6C). We found that overexpression of p300WT reduced basal reporter activity by 47% (Fig. S4). These data support our p300 RNAi results showing that p300 has a repressive role in basal DUSP1 gene expression. In contrast to p300WT, overexpression of p300ΔHAT reduced basal reporter activity approximately 24% (Fig. S4). Overexpression of p300WT potentiated the DEX-induced reporter gene expression response from 5.8 fold to 9.5 fold (Fig. 6C), whereas overexpression of p300ΔHAT increased the DEX response to 7.8 fold (Fig. 6C). These data confirm that in response to glucocorticoids, the mechanism of p300 coactivation of DUSP1 gene transcription involves, but is not limited to, p300 HAT activity.

Finally, we performed ChIP-reChIP experiments to determine if p300 and GR are recruited to the same genomic region of DUSP1. GR antibodies were first used to pull down GR-associated DNA fragments. Antibodies against p300 were then used to isolate GR-associated DNA fragments that also associate with p300. Compared to ChIP-reChIP using IgG control antibodies, ChIP-reChIP using GR and then p300 showed significant enrichment of the DUSP1 GRE (23 fold, Fig. 6D). These results suggest that GR and p300 are in the same protein complex recruited to the DUSP1 GRE.

## Discussion

Multiple mechanisms contribute to the anti-inflammatory effects of glucocorticoids. One of them is the ability of glucocorticoids to induce the transcription of DUSP1. Thus, understanding the regulatory mechanism of glucocorticoid-activated DUSP1 gene transcription is vital for future therapeutic interventions against inflammatory diseases. In this study, we systematically investigated the mechanisms of GR-regulated DUSP1 gene transcription. First, we determined that GR regulates the expression of DUSP1 through transcriptional initiation (Fig. 1A); then, we identified the GRE at the human DUSP1 promoter that is responsible for this regulation (Fig. 1B, 1C). The GRE appeared to be in organized nucleosomal structures, within which GR is bound at a consensus GR binding site (GLS2) at the GRE (Fig. 2 and Fig. 3). We also showed that the induction of DUSP1 gene transcription by glucocorticoids involves the opening up of chromatin structure in the DUSP1 promoter region corresponding with changes in the nucleosomal status (Fig. 3). While histone H3 and H4 of the DUSP1 promoter are already acetylated in the basal state, glucocorticoids further increased acetylated histone H3 and H4 levels (Fig. S2 and Fig. 4). Finally, we showed that p300 acts as a transcriptional coactivator for GR in the induction of DUSP1 gene transcription (Fig. 5 and Fig. 6). GR and p300 appear to be in the same protein complex recruited to the DUSP1 GRE (Fig. 6D), and reducing the expression of p300 in cells specifically decreased glucocorticoid-stimulated histone H3 acetylation (Fig. 6A). Overall, these observations provide a solid foundation that will allow us to further dissect this important transcriptional regulatory mechanism.

A recent study using genomic conservation analyses identified multiple GREs upstream of the human DUSP1 gene [31]. These GREs include the one reported here, located ∼1.3 kb upstream of the TSS. In a reporter gene that contains this GRE and another located 4.6 kb upstream of the TSS, Tchen et al. showed that the mutation of either GRE reduces hormonal response by approximately 50%. Thus, both GREs play a role in glucocorticoid-regulated DUSP1 gene transcription. This confirms the importance of our studies on the role of the presently characterized GRE in overall transcriptional activation of the DUSP1 gene. The locations of other GREs identified by Tchen et al. are far away from the TSS (29, 28 and 24 kb), and genome-wide analysis of GR binding regions indeed reveals that many potential GREs are located far away from the TSS [32], [33]. However, the importance of these long-range GREs in DUSP1 gene regulation is unclear at the moment, as these GREs have not been studied in their natural configuration. Future studies should address these questions.

In addition to GLS2, we identified three other GLS elements that may play accessory roles in the regulation of DUSP1 gene transcription. Interestingly, only one of these three is required for a complete glucocorticoid response, and any of these three can play the accessory role. It is unclear whether the mechanisms governing the functional interactions between GLS2 and GLS1, 3, or 4 are the same. This will require detailed studies of the role of these accessory elements in glucocorticoid response. At present, we cannot be sure if they directly associate with GR. It is possible that GLS2, which fits the consensus sequence of a canonical GR binding site, serves as an essential site for the binding of GR while the other GLS elements, which are more divergent, can only bind to the receptor when GR:GLS2 interacts. Alternatively, these GLS elements may bind to other transcriptional regulators. The requirement of functional interactions between GR and other DNA-binding transcriptional regulators to confer a complete glucocorticoid response has been previously reported. These functional interactions are required in GREs of rat PEPCK [34], [35], [36], [37], rat tyrosine aminotransferase (TAT) [38], [39] and rat glucose-6-phosphatase [40] genes. Thus, these GLS elements could add another layer of control to the regulatory mechanisms of GR-activated DUSP1 gene expression. Notably, within the GRE identified here, there is a CCAAT enhancer binding protein (C/EBP) site (−1312 and −1305) [41], located adjacent to the GR binding sequence (−1337 and −1323). This configuration of the DUSP1 GRE is reminiscent of glucocorticoid-activated rat α1-acid glycoprotein (AGP) in hepatocytes. The rat AGP gene GRE is a composite response element that contains both GR and C/EBPβ binding sites – requiring both for synergistic activation [42], [43]. Intriguingly, transcriptional synergism is still observed even when one of the two factors lacked either its DNA-binding or transcriptional-activation function. Thus, a direct protein-protein interaction between these two distinct transcription factors is probably required to regulate the rat AGP gene [42], [43]. Supporting this mechanism, overexpression of a GR mutant defective in DNA binding still potentiates the glucocorticoid response in DUSP1 gene expression [41]. The functional interaction between GR and C/EBPβ has also been reported in the regulation of other glucocorticoid target genes, such as phosphoenolpyruvate carboxykinase (PEPCK) and β-casein [44], [45]. The role of C/EBP binding site in glucocorticoid-activated DUSP1 gene transcription merits future studies.

Chromatin structure plays a critical role in the regulation of gene transcription. Based on our findings from DNase I accessibility experiments, the DUSP1 genomic region up to ∼2 kb upstream of the TSS is already opened up prior to treatment with glucocorticoids (Fig. 3A). Glucocorticoid treatment further increases this region's DNase I accessibility except in the area from −160 to −64. The results from MNase experiments indicate that nucleosomes 1 and 2 are likely remodeled, increasing the accessibility of DNase I to chromatin in this region. Previous studies have shown that transcriptional activation of several glucocorticoid target genes requires the SWI/SNF chromatin remodeling complex [46], [47]. When we knocked down the expression of Brm, an ATPase subunit of SWI/SNF, in A549 cells, the ability of glucocorticoids to stimulate several of their target genes was reduced – intriguingly, DUSP1 escapes this requirement for Brm (data not shown). This suggests that Brg1, another ATPase component of the SWI/SNF complex, may compensate for the loss of Brm in A549 cells. Alternatively, it is possible that chromatin remodeling complexes other than SWI/SNF are involved in glucocorticoid-regulated DUSP1 gene transcription. We are currently investigating these possibilities.

The analyses of histone acetylation status in the DUSP1 gene promoter showed that the genomic region up to 2 kb upstream of the TSS is already acetylated in its basal state. These results are in agreement with the DNase I accessibility findings discussed above and are not surprising, as DUSP1 gene expression is not silent in the absence of glucocorticoid treatment. The fact that glucocorticoid treatment further increases the acetylation status of histone H3 and H4 indicates that HAT(s) are correlated with the glucocorticoid response on DUSP1 gene expression. We found two HATs, p300 and CBP, whose recruitment to the DUSP1 GRE is increased upon DEX treatment. RNAi knockdown of CBP fails to affect basal or glucocorticoid-stimulated DUSP1 gene expression, both of which are affected by p300 RNAi (Fig. 5C and Fig. S3). Although p300 and CBP frequently exert redundant functions in vitro, their roles in DEX-activated DUSP1 gene transcription may be distinct. Alternatively, the CBP protein remaining after RNAi knockdown may be sufficient to maintain normal effects of CBP on DUSP1 gene expression. While further investigation is necessary to examine these possibilities, our current study better illuminates the role of p300.

We found that p300 is present in the DUSP1 gene promoter in the basal state, and glucocorticoid treatment further augments its occupancy. It is possible that p300 is recruited to the promoter region near the DUSP1 GRE through existing DNA-binding transcriptional regulators. Upon glucocorticoid treatment, GR binds to the GRE and further recruits p300 to the GRE. Interestingly, the results from our RNAi experiments indicate that p300 has dual roles on DUSP1 gene expression. RNAi knockdown of p300 increases basal gene expression of DUSP1, but also limits the ability of glucocorticoids to potentiate DUSP1 gene transcription. Thus, p300 likely acts as a corepressor for basal DUSP1 expression, but as a coactivator for GR-stimulated DUSP1 gene transcription. Other results in this report support this model. Supporting the corepressor role, p300 overexpression reduced the activity of a reporter gene containing the −1672 to −959 region of DUSP1 gene. This suggests that the element(s) responsible for repressive effects of p300 is located in this genomic region. Notably, it has been reported that p300 can reduce the transcriptional activity of certain DNA-binding transcription factors [48], [49], and it will be interesting to identify these element(s) and their associated proteins in future studies. Supporting the coactivator role of p300, overexpression of p300 increased DEX-induced reporter activity from 5.8 to 9.5 fold, whereas a p300 mutant lacking HAT activity only stimulated the reporter activity to 7.8 fold. Together, these results indicate that the HAT activity of p300 plays a role in DEX-induced DUSP1 gene expression. RNAi knockdown of p300 in A549 cells abolished DEX-induced H3 acetylation, which further supports the role of HAT activity in p300 but does not exclude other potential mechanisms of p300 activity. Finally, ChIP-reChIP experiments showed GR and p300 are in the same protein complex that associates with the DUSP1 GRE, which also supports the role of p300 as a GR coactivator.

Glucocorticoids are an essential tool in combating inflammatory disorders, however their use is limited by multiple serious side effects. There is a conception that glucocorticoids' anti-inflammatory effects are attributed to GR-regulated transcriptional repression, while the side effects reflect transcriptional activation by GR. However, recent studies clearly demonstrate the importance of glucocorticoid-activated DUSP1 gene expression in mediating the anti-inflammatory effects of glucocorticoids. Thus, an important topic in glucocorticoid pharmacology will be how to dissociate transcriptional activation of GR-induced anti-inflammatory actions from the undesired side effects. To do this, it is critical to identify the specific components of the mechanisms governing GR-activated transcription of DUSP1 and other genes involved in the induction of unwanted side effects.

## Supporting Information

Figure S1 DNaseI accessibility of α-fetoprotein gene in response to glucocorticoids. An example of a gene whose chromatin structure is not opened up in response to glucocorticoids. α-fetoprotein (AFP) gene schematic shows the location of three primers used (black boxes labeled A1–A3) for qPCR analyses. The regions of the AFP gene (relative to the TSS) amplified by these primers are: A1 (−729 to −652), A2 (−405 to −324), and A3 (−183 to −99). DNase I accessibility assay and calculations were performed as described in Figure 3. Data represent the SEM of percentage increase (DNaseI-cut cells divided by uncut cells) from at least three experiments.
(0.29 MB TIF)

Figure S2 Basal and glucocorticoid-induced histone H3 and H4 acetylation of the DUSP1 gene promoter. A AcH3/H3 ChIP experiments presented in Figure 4A, with results shown relative to IgG ChIP. B AcH4/H4 ChIP experiments presented in Figure 4B, with results shown relative to IgG ChIP. The data represent the SEM of the fold induction (DEX-treated cells divided by DMSO-treated cells) from at least four experiments.
(0.45 MB TIF)

Figure S3 Effects of p300 and CBP siRNA on basal DUSP1 gene expression. RNAi knockdown experiments presented in Figure 5C, showing RNAi against p300 and CBP increased basal (DMSO-treated) DUSP1 gene expression relative to scramble (control). Data represent the SEM of fold induction (p300 or CBP siRNA divided by scramble control) from at least three experiments.
(0.16 MB TIF)

Figure S4 Effects of p300WT and p300ΔHAT overexpression on basal activity of pDUSP1 reporter gene. pDUSP1 reporter assay presented in Figure 6C, showing both pCI-p300WT and pCI-p300ΔHAT decreased basal (DMSO-treated) reporter activity. Data represent the SEM of fold induction (pCI-p300WT or pCI-p300ΔHAT divided by pCI control) from five experiments.
(0.15 MB TIF)

Table S1 Primers.
(0.04 MB DOC)
