# Supplementary figures and images for: Transcriptional Regulation of Human Dual Specificity Protein Phosphatase 1 (DUSP1) Gene by Glucocorticoids
Source: PLoS One. 2010 Oct 29;5(10):e13754. doi: 10.1371/journal.pone.0013754 (PMC2966426; doi:10.1371/journal.pone.0013754)

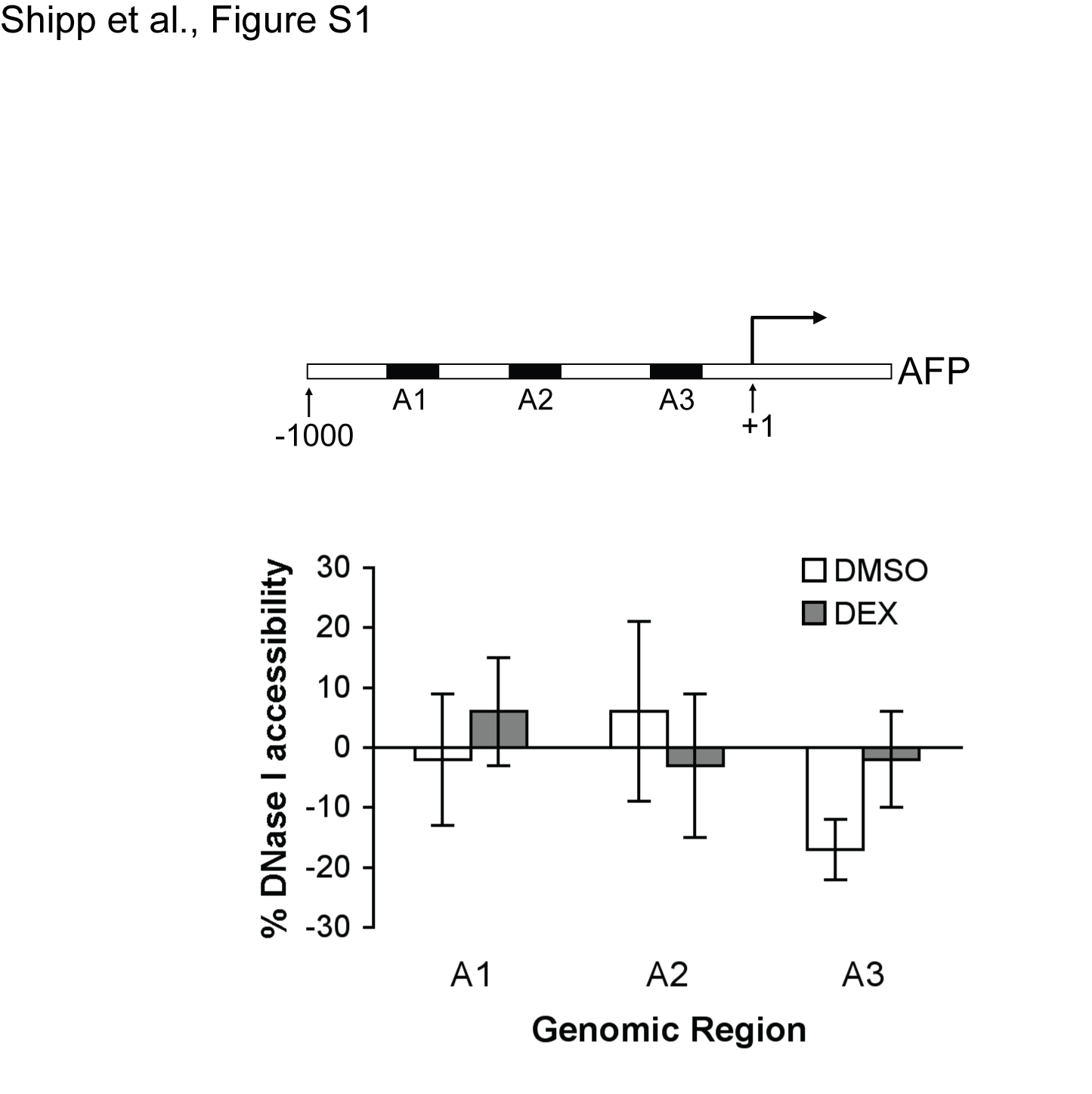

Supplement: Figure S1 — DNaseI accessibility of α-fetoprotein gene in response to glucocorticoids. An example of a gene whose chromatin structure is not opened up in response to glucocorticoids. α-fetoprotein (AFP) gene schematic shows the location of three primers used (black boxes labeled A1–A3) for qPCR analyses. The regions of the AFP gene (relative to the TSS) amplified by these primers are: A1 (−729 to −652), A2 (−405 to −324), and A3 (−183 to −99). DNase I accessibility assay and calculations were performed as described in Figure 3. Data represent the SEM of percentage increase (DNaseI-cut cells divided by uncut cells) from at least three experiments. (0.29 MB TIF) [file pone.0013754.s001.tif]

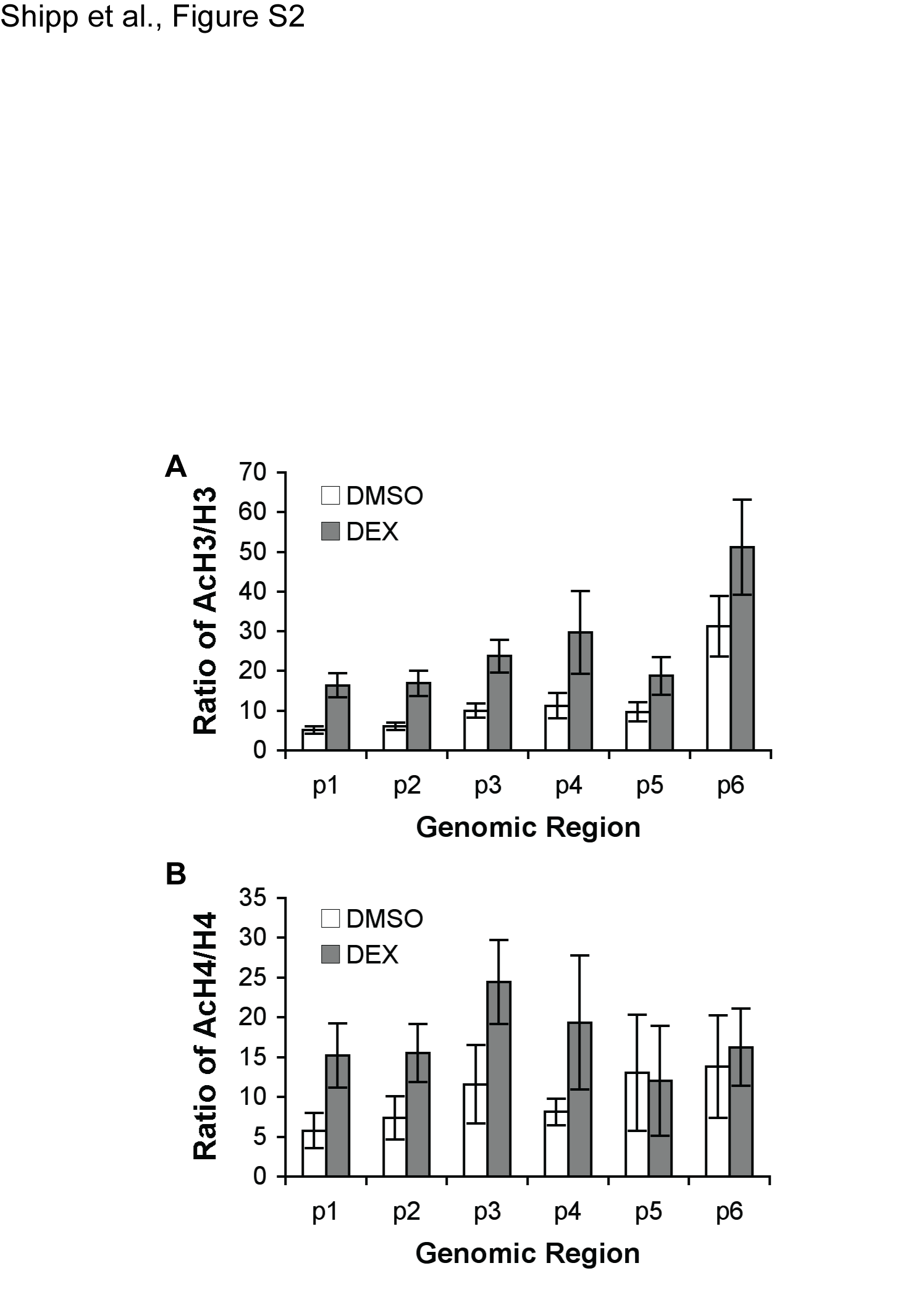

Supplement: Figure S2 — Basal and glucocorticoid-induced histone H3 and H4 acetylation of the DUSP1 gene promoter. A AcH3/H3 ChIP experiments presented in Figure 4A, with results shown relative to IgG ChIP. B AcH4/H4 ChIP experiments presented in Figure 4B, with results shown relative to IgG ChIP. The data represent the SEM of the fold induction (DEX-treated cells divided by DMSO-treated cells) from at least four experiments. (0.45 MB TIF) [file pone.0013754.s002.tif]

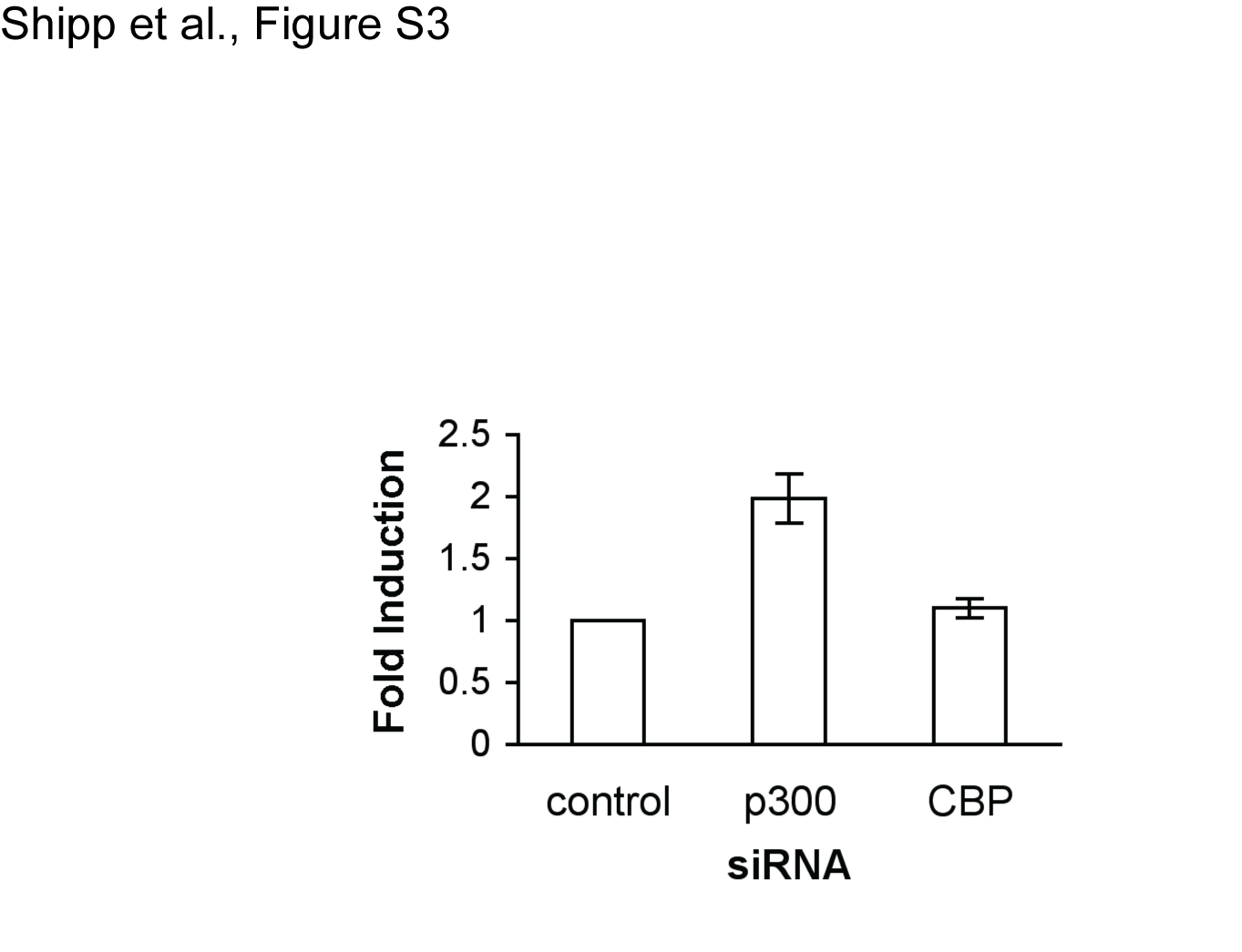

Supplement: Figure S3 — Effects of p300 and CBP siRNA on basal DUSP1 gene expression. RNAi knockdown experiments presented in Figure 5C, showing RNAi against p300 and CBP increased basal (DMSO-treated) DUSP1 gene expression relative to scramble (control). Data represent the SEM of fold induction (p300 or CBP siRNA divided by scramble control) from at least three experiments. (0.16 MB TIF) [file pone.0013754.s003.tif]

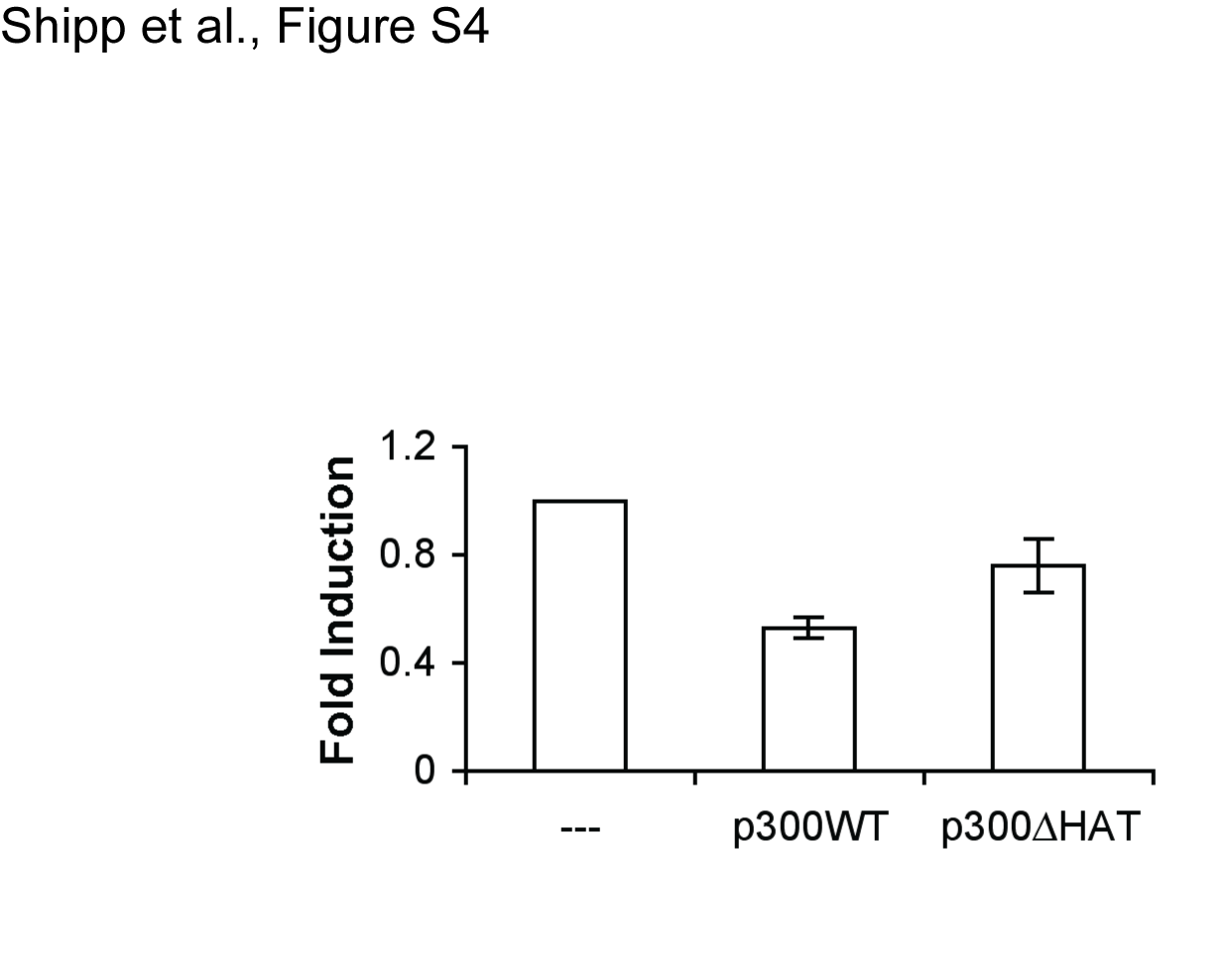

Supplement: Figure S4 — Effects of p300WT and p300ΔHAT overexpression on basal activity of pDUSP1 reporter gene. pDUSP1 reporter assay presented in Figure 6C, showing both pCI-p300WT and pCI-p300ΔHAT decreased basal (DMSO-treated) reporter activity. Data represent the SEM of fold induction (pCI-p300WT or pCI-p300ΔHAT divided by pCI control) from five experiments. (0.15 MB TIF) [file pone.0013754.s004.tif]
