# Supplementary material for: Transcriptional Regulation of Human Dual Specificity Protein Phosphatase 1 (DUSP1) Gene by Glucocorticoids
Source: PLoS One. 2010 Oct 29;5(10):e13754. doi: 10.1371/journal.pone.0013754 (PMC2966426; doi:10.1371/journal.pone.0013754)
Supplement: Table S1 — Primers. (0.04 MB DOC) [file pone.0013754.s005.doc]

Table S1

Primers used for MNase experiments

hMKP1/-1141/Mnase/fo GGATTTTGCTTTCGGCCTAT

hMKP1/-1091/Mnase/re ACAACCCTCGCTCCCTGT

hMKP1/-1243/Mnase/fo CCGTCACTGGGACTCAGG

hMKP1/-1181/Mnase/re CGCAACACTTGGGAAGACC

hMKP1/-1428/Mnase/fo CAGAAGTTGCCACTGGTGAT

hMKP1/-1375/Mnase/re GCTGGCCACCCTGTGT

hMKP1/-1580/Mnase/fo TGAGGACCTCTTTGCTGTCC

hMKP1/-1525/Mnase/re AAGGGCGTCTTCTACCTTCA

hMKP1/-1544/Mnase/fo TGAAGGTAGAAGACGCCCTTT

hMKP1/-1492/Mnase/re AAAGTTCAGGGCATGGAGTG

hMKP1/-1484/Mnase/fo AGAATCCAGGCAGAACATTTG

hMKP1/-1433/Mnase/re GCCTCGCTTAGCTTGTGTGT

hMKP1/-1414/Mnase/fo TGGTGATACAGCTCGcaca

hMKP1/-1356/Mnase/re CTGGGGGAGGGGATTTC

hMKP1/-1371/Mnase/fo AATCCCCTCCCCCAGGA

hMKP1/-1321/Mnase/re TGCCGAGTCAGGAACATTCT

hMKP1/-1344/Mnase/fo CGCAGAATCTTCCTGACTCG

hMKP1/-1285/Mnase/re GGGTACACAAACATAAACAATGC

hMKP1/-1266/Mnase/fo CAGCAGGCTCCGCTGT

hMKP1/-1215/Mnase/re ATCTCCGTGCCCTGAGTC

hMKP1/-1229/Mnase/fo CAGGGCACGGAGATCG

hMKP1/-1179/Mnase/re ATCGCAACACTTGGGAAGAC

hMKP1/-1167/Mnase/fo CAATCCGCCCACAATGG

hMKP1/-1118/Mnase/re CGTTATAGGCCGAAAGCAAA

hMKP1/-1137/Mnase/fo TTTGCTTTCGGCCTATAACG

hMKP1/-1078/Mnase/re ACAGAAGCCGGCCACA

hMKP1/-1084/Mnase/fo CTTCTGTTCCGGGTTGGAG

hMKP1/-1035/Mnase/re GTGAGCGCCCACTAAGC

Primers used for nuclear run-on

hDUSP1-run on-left TTGCCCACACCAGACTGATA

hDUSP1-run on-right AGGACAACCACAAGGCAGAC

hDUSP1-run on-2-left CAGGTACAGAAAGGGCAGGA

hDUSP1-run on-2-right CAGGCAAATGGGCTTAGTTC

hbeta-actin-run-on-left GGGGTGTTGAAGGTCTCAAA

hbeta-actin-run-on-right TCTGAACAGACTCCCCATCC

Primers used to detect MKP-1 expression

hDUSP1-602-fo CTGCCTTGATCAACGTCTCA

hDUSP1-696-re CTGTGCCTTGTGGTTGTCCT

Primers used for site-directed mutagenesis

hDUSP1-pGRE4m-Fo CAATCCGCCCACAATTGCCCGGGATTGG

hDUSP1-pGRE4m-Re CCAATCCCGGGCAATTGTGGGCGGATTG

hDUSP1-pGRE1m-Fo CGACGACACAGGGTTGCCAGCGAAATCC

hDUSP1-pGRE1m-Re GGATTTCGCTGGCAACCCTGTGTCGTCG

hDUSP1-pGRE2m-Fo GAGGAAACCGCAGAATTTTCCTGACTCGGCAC

hDUSP1-pGRE2m-Re GTGCCGAGTCAGGAAAATTCTGCGGTTTCCTC

hDUSP1-pGRE3m-Fo GCAGGCTCCGCTTTCCAGGGGGCCG

hDUSP1-pGRE3m-Re CGGCCCCCTGGAAAGCGGAGCCTGC

hDUSP1-pGRE5m-Fo GCTTAGTGGGCGCTGACTGTGTATACTGCC

hDUSP1-pGRE5m-Re GGCAGTATACACAGTCAGCGCCCACTAAGC

Primers used for ChIP-scanning and DNase I accessibility

hDUSP1/-1815/ChIP-Fo GCGCCCAGCTCTTAAAAAGT

hDUSP1/-1717/ChIP-Re CCGACTTGATTTGTCCCATT

hDUSP1/-662/ChIP-Fo GCTCGAGTCGGTCTTGGTAG

hDUSP1/-543/ChIP-Re GACTTGCCCAGAACCACACT

hDUSP1/-106/ChIP-Fo CCGTCACGTGATCACCATT

hDUSP1/-16/ChIP-Re GCGTTTATATGCGGCCTCT

hDUSP1/+98/ChIP-Fo ACGCTCCTCGCTCAGTCCAA

hDUSP1/+215/ChIP-Re GCGGTGCTCTTTGTCTGTTC
